# Editorial: The gut-pancreas axis in type 1 diabetes – a focus on environmental factors

**DOI:** 10.3389/fendo.2023.1270297

**Published:** 2023-08-15

**Authors:** Tina Fløyel, David Funda, Veronica I. Dodero, Martin Haupt-Jorgensen

**Affiliations:** ^1^ Translational Type 1 Diabetes Research, Clinical Research, Steno Diabetes Center Copenhagen, Herlev, Denmark; ^2^ Institute of Microbiology of the Czech Academy of Sciences, v.v.i., Prague, Czechia; ^3^ Department of Chemistry, Organic Chemistry III, Bielefeld University, Bielefeld, Germany; ^4^ The Bartholin Institute, Department of Pathology, Rigshospitalet, Copenhagen, Denmark

**Keywords:** type 1 diabetes, environmental factors, gluten-free diet, enterovirus, gut-pancreas axis, NOD mouse, beta-cell stress, intestinal permeability

The global type 1 diabetes (T1D) incidence is rising too fast to be explained by genetic drift, thereby underlining the importance of environmental factors. Drawing further attention to environmental factors as being responsible for the rising T1D incidence is the reduced occurrence of high-risk haplotypes within the HLA genes in individuals developing T1D compared to previously (1). Several environmental factors have been associated with T1D, primarily from work on the non-obese diabetic (NOD) mouse model, and some of these factors, e.g. gluten (2) and enterovirus (3), have led to human intervention trials. Many of the proposed food and microbial environmental factors can travel to the pancreatic islets from the intestinal lumen after crossing the intestinal barrier (4). In the pancreatic islets they are hypothesized to contribute to autoimmunity, e.g. *via* induction of beta-cell stress. Thus, the intestinal barrier function is likely of key importance. Noteworthy, intestinal permeability is increased both in pre- and clinical T1D (5), likely permitting luminal environmental factors easier entry. The present Research Topic gives an update on the involvement of environmental factors and the gut-pancreas axis in T1D.

Several of the Research Topic aspects are described in the review by Buschard, dealing with possible causes and interventions for T1D. The review discusses the involvement of the intestine in T1D. It highlights both the mucin degrading bacterium *Akkermansia Muciniphila*, which can decrease the intestinal permeability, and gluten, which has been shown to increase intestinal permeability *via* the enterocyte chemokine receptor CXCR3. Interestingly, both *Akkermansia Muciniphila (* 6) and gluten-free diet (7) have been shown to delay diabetes development in NOD mice, pointing to future treatment targets. Regarding gluten-free diet, originally no beneficial effects were reported (8, 9), however, a more recent pilot study and a human intervention trial documented beneficial effects in new-onset T1D children (2, 10), thus more research is needed. Another theme in the review deals with beta-cell stress, which according to Buschard could mediate formation of immunogenic insulin molecules and autoimmunity. The section on sphingolipids mostly concerns sulfatide, which is both present in beta cells and nerve myelin sheets but with different functions, namely folding of insulin and facilitation of electric impulses, respectively. Interestingly, the level of sulfatide is substantially lower in individuals with new-onset T1D and could therefore be an early treatment target in T1D.

The intestinal microbiota is skewed in T1D (11) and, as discussed in the review by Buschard, the gut barrier integrity is influences by some bacteria. The original paper by Luo et al. examined the intestinal microbiota and serum metabolite composition in 49 and 52 T1D patients positive and negative for glutamic acid decarboxylase antibody (GADA+/-), respectively. The authors found that the intestinal microbiome and serum metabolite profiles differed between the GADA+/- patients. Furthermore, the abundance of the bacterial genus *Alistipes* was negatively associated with serum metabolites involved in tryptophan metabolism, meaning that the microbiota changes in the GADA+ T1D patients may contribute to lower tryptophan-related metabolites. This is an interesting finding, as tryptophan metabolites can bind to the aryl hydrocarbon receptor, resulting in secretion of interleukin 22, improved intestinal barrier, increased gastrointestinal motility, anti-inflammatory properties, and modulation of intestinal microbiota (12–14).

Enterovirus may be a trigger of T1D. This is primarily based on the detection of enterovirus in pancreatic islets from new-onset T1D patients (15, 16). The original article by Josefsen et al. deals with the question why 30% of the beta cells are inactive at T1D diagnosis and how they can be activated, thus relieving the stress on the active beta cells. Using DiViD and nPOD tissues, the authors analyzed gene expression levels in islets from individuals at different T1D stages. The main findings in the T1D islets were changes in genes associated with fetal dedifferentiation and asynchrony, which could explain the inactivity of the beta cells at diagnosis. The authors propose to treat T1D patients with type 2 diabetes drugs, such as GLP-1 receptor agonists and metformin, combined with anti-inflammatory compounds, to activate the inactive beta cells and prevent autoimmunity. The analyses in the article by Josefsen et al. were done on whole islets, so it is unknown which islet cell type(s) are responsible for the authors observations of modulated gene expressions. Previous studies show mixed results of treating T1D patients with GLP-1 receptor agonists (Exenatide, Albiglutide) (17–19), although a combination therapy with anti-inflammatory compounds is, to our knowledge, unexplored.

To investigate how environmental factors affect the pancreatic beta cells it is crucial with a beta-cell model that closely resembles native human beta cells. In the original article by Frørup et al. results of an in-depth characterization of the effects of pro-inflammatory cytokines on EndoC-βH5 cells are presented. This new non-proliferative beta-cell model has the advantage of being non-cancerous, free of xenotropic murine virus, and with high insulin secretion capacity. The authors report that EndoC-βH5 cells are particularly responsive to interferon-γ over other diabetogenic cytokines, which is not necessarily comparable to native human beta cells, resulting in upregulation of key cellular responses, such as MHC-I. Further studies of EndoC-βH5 cells are required, but Frørup et al. contributes with important new insights into this promising new cell model.

In conclusion, this Research Topic provides encouraging new data and hypotheses on several etiopathogenetic areas in T1D. This includes data indicating why a high percentage of beta cells are inactive at T1D diagnosis as well as microorganisms and metabolites that may play a role in intestinal barrier dysfunction in T1D. The interplay of environmental factors, followed by possible changes in the intestinal permeability and/or mucosal immune mechanisms are complex and difficult to dissect, e.g. both gut microbiota-dependent and independent dietary effects were reported in NOD mice (20, 21). Nevertheless, the underlying feature of these factors seems linked to the persistence of chronic intestinal inflammation (22). More work is needed to identify the environmental factors behind the rising T1D incidence, the time windows critical for the exposure of such factors, how they influence the intestinal barrier function, the underlying mechanisms of the elicitation of autoimmunity, and which interventions can prevent and treat T1D.

## Author contributions

TF: Writing – review & editing. DF: Writing – review & editing. VD: Writing – review & editing. MH-J: Writing – original draft, Writing – review & editing.
